# Subcallosal Cingulate Cortex Deep Brain Stimulation for Treatment-Resistant Depression: A Systematic Review

**DOI:** 10.3389/fneur.2022.780481

**Published:** 2022-04-01

**Authors:** Michał Sobstyl, Anna Kupryjaniuk, Marek Prokopienko, Marcin Rylski

**Affiliations:** ^1^Department of Neurosurgery, Institute of Psychiatry and Neurology, Warsaw, Poland; ^2^Department of Radiology, Institute of Psychiatry and Neurology, Warsaw, Poland

**Keywords:** deep brain stimulation, major depressive disorder, treatment-resistant depression, subcallosal cingulate cortex, depression

## Abstract

**Background:**

Deep brain stimulation (DBS) is considered a relatively new and still experimental therapeutic modality for treatment-resistant depression (TRD). There is clinical evidence to suggest that stimulation of the subcallosal cingulate cortex (SCC) involved in the pathogenesis of TRD may exert an antidepressant effect.

**Aims:**

To conduct a systematic review of current studies, such as randomized clinical trials (RCTs), open-label trials, and placebo-controlled trials, examining SCC DBS for TRD in human participants.

**Method:**

A formal review of the academic literature was performed using the Medical Literature, Analysis, and Retrieval System Online (MEDLINE) and Cochrane Central Register of Controlled Trials (CENTRAL) databases. This systematic review was conducted in accordance with the Preferred Reporting Items for Systematic Reviews and Meta-Analyses (PRISMA) guidelines. Suitable studies were screened and assessed based on patient characteristics, clinical outcomes, adverse events related to DBS, and the stereotactic technique used to guide the implantation of DBS electrodes.

**Results:**

The literature search identified 14 clinical studies that enrolled a total of 230 patients with TRD who underwent SCC DBS. The average duration of follow-up was 14 months (range 6–24 months). The response and remission rates at the last available follow-up visit ranged between 23–92% and 27–66.7%, respectively.

**Conclusion:**

The current results of SCC DBS are limited by the relatively small number of patients treated worldwide. Nevertheless, studies to date suggest that SCC can be a promising and efficacious target for DBS, considering the high response and remission rates among patients with TRD. The adverse events of SCC DBS are usually transient and stimulation-induced.

## Introduction

Major depressive disorder (MDD) is one of the most common psychiatric diseases and a leading cause of disability worldwide (1). The prevalence of MDD in the United States of America is estimated at 5–8% (2). MDD is a lifelong disorder characterized by symptoms that have a debilitating impact on the patient's daily life (3). The defining symptoms of MDD include a depressed mood, decreased energy, anhedonia, insomnia or hypersomnia, and psychomotor agitation or retardation (3). MDD can cause difficulties in daily functioning by decreasing the ability to maintain a job, perform daily activities, or function in society and is also associated with a significant risk for suicide (4). It also contributes to an increased risk of cardiovascular disease and stroke and may exacerbate the course of other diseases. In addition to its negative effects on the individual patient, MDD also imposes a significant public healthcare burden (5). Either “depressed mood” or “loss of interest or pleasure” is essential for a diagnosis of MDD (6).

There are several non-invasive and effective treatments available for MDD. The most common conventional MDD treatments are pharmacotherapy, psychotherapy, such as cognitive-behavioral therapy (CBT), and electroconvulsive therapy (ECT) (6). Although there are many patients who initially show a favorable response to treatment, there is a significant percentage of patients who fail to respond, resulting in an estimated 1–3% prevalence of treatment-resistant depression (TRD) (7). TRD is associated with more comorbid mental disorders, a higher number of hospitalizations, and a high rate of suicidal attempts (30%) (7, 8). Patients with TRD also represent a significant portion of the demand for emerging non-pharmacological treatment options, such as repetitive transcranial magnetic stimulation (rTMS) and vagus nerve stimulation (VNS) (9, 10). A promising but highly invasive neurosurgical option for TRD patients is deep brain stimulation (DBS) (11).

The aim of this systematic review was to provide a detailed description of clinical studies which examined the role of subcallosal cingulate cortex (SCC) DBS in patients with TRD. The neuroanatomical connections of the SCC are described, with its pivotal role in the pathogenesis of depression. The safety profile of SCC DBS is also discussed, with a focus on the most common stimulation-induced adverse events.

## Materials and Methods

### Selection of SCC DBS Studies for TRD

A systematic review was conducted in order to select suitable studies examining SCC DBS in TRD published between January 2005 and January 2021. The search algorithm included the following search terms: “deep brain stimulation,” “major depressive disorder,” “treatment-resistant depression,” and “subcallosal cingulate cortex.” The following electronic databases were searched: Medical Literature, Analysis, and Retrieval System Online (MEDLINE) and the Cochrane Central Register of Controlled Trials (CENTRAL). The literature search was performed in accordance with the recommendations outlined in the Preferred Reporting Items for Systematic Reviews and Meta-Analyses (PRISMA) guidelines. We limited our search to clinical studies, which enrolled human participants, irrespective of design, and considered research articles published in English. DBS studies were included if the sample included at least five patients with TRD. This limit was imposed because studies, which included fewer than five patients often reported individual patient outcomes, rather than looking at data for the whole sample. In addition, with small sample sizes, the presence of outliers can significantly affect data analysis. A placebo effect is very strong for all functional neurosurgical procedures, especially in the field of neuropsychiatry. In order to minimize its impact on the final clinical outcomes, a minimum postoperative follow-up period of 6 months was chosen.

**Figure 1:**
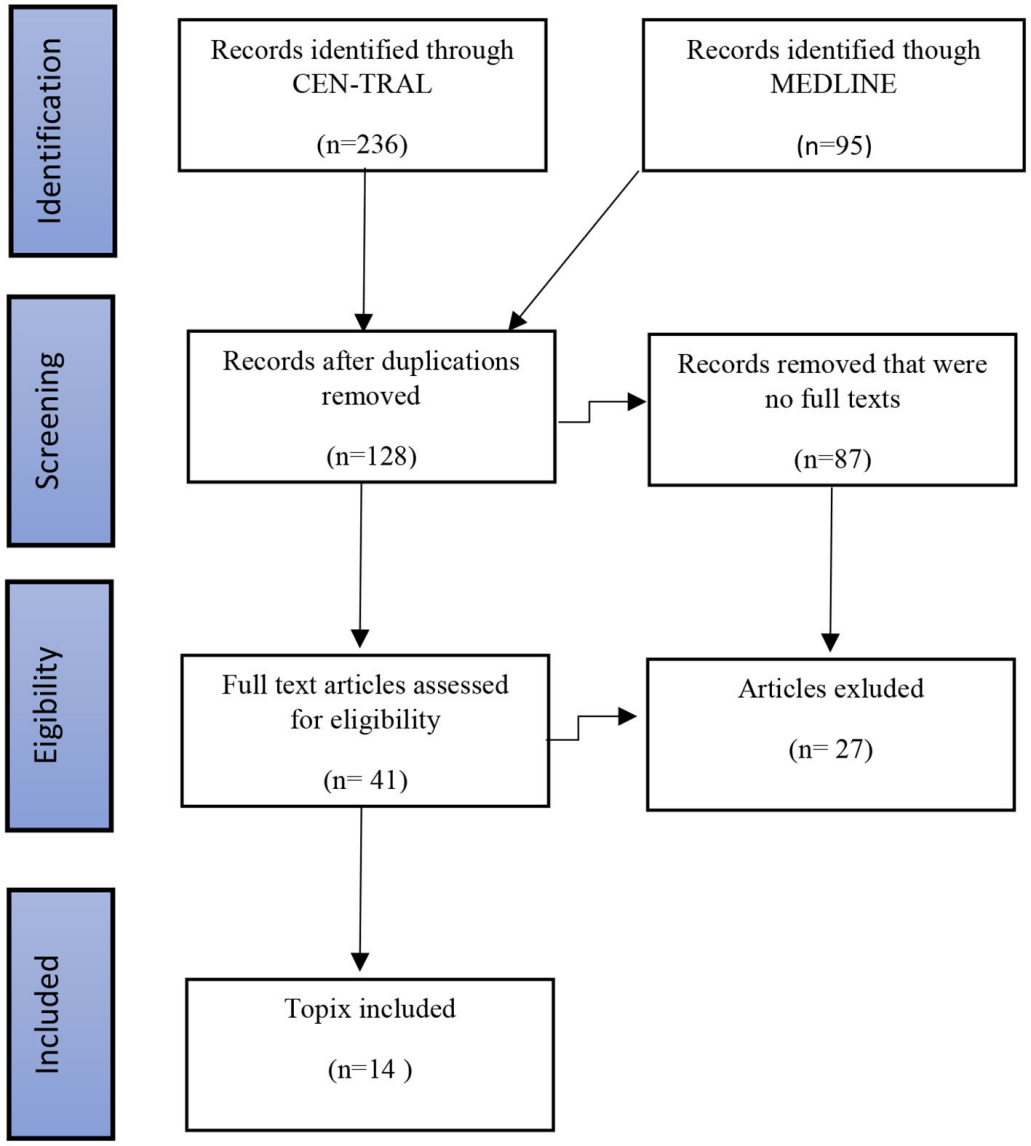
Procedures followed for the identification of eligible studies examining SCC DBS for TRD using the PRISMA guidelines.

Exclusion criteria were as follows: animal studies; studies, which enrolled TRD patients not treated with DBS; pre-clinical studies; case reports, such as those focused on neurosurgical techniques; review articles; and letters to the editor. We also excluded studies which described duplicate cohorts, clinical studies enrolling less than five patients, or those with a follow-up period shorter than 6 months. The procedures for selecting the final articles following a search of the academic databases, based on our inclusion and exclusion criteria, and using the PRISMA guidelines, are detailed in Figure 1. The studies selected for inclusion in our review (*n* = 14) articles all examined the use of SCC DBS in the management of TRD.

### The Pathophysiology of MDD

MDD is considered a neuropsychiatric disorder caused by dysfunction of the limbic cortico-striato-thalamo-cortical (CSTC) mood circuit (12, 13). Based on this model, three main components of the CSTC mood circuit are proposed. First, the ventral component that comprises the amygdala, ventral striatum (VS), which includes the nucleus accumbens (NAc) and olfactory tubercle, and the orbitofrontal cortex (OFC), the ventral part of the anterior cingulate cortex (ACC), the ventrolateral prefrontal cortex (VLPFC), and downstream structures that include the lateral hypothalamus (LH), and locus coeruleus (LC). The ventral component of the CSTC also includes the SCC, which projects to the amygdala, hippocampus, superior and medial temporal gyri, NAc, posterior cingulate cortex, thalamus, hypothalamus, periaqueductal gray matter, and lateral habenula. The ventral component of the CSTS mood circuit, in particular, the NAc, mediates reward, cognition, reinforcement, and motivational salience. The ventral component of the CSTS is responsible for emotional recognition and an adequate emotional and behavioral response (12–14).

The dorsal component of the CSTC mood circuit comprises the dorsolateral (dlPFC) and dorsomedial prefrontal (dmPFC) cortices, a dorsal part of the ACC, and the hippocampus. The dorsal component of the CSTC is essential for regulating emotional responses, cognition, and motor and certain executive functions. The third component is thought to comprise a modulating region restricted to the thalamus and the rostral ACC. There is a small degree of overlap between the ventral and dorsal striatum, which is also a component of a reward system that, along with the NAc, mediates the encoding of new motor programs associated with future reward acquisition (12–14).

### The SCC as a Target for TRD

Mayberg proposed a model whereby MDD is associated with decreased activity in the dorsal limbic and neocortical regions and with increased activity in ventral limbic and paralimbic structures (13). Positron emission tomography (PET) studies have provided accumulating evidence that hyperactivity is observed, especially in the SCC, in individuals experiencing sadness, or patients with untreated MDD (15). Neuronal connections of the SCC correspond to its involvement in the large-scale neuronal network dysfunction in TRD. The SCC contains three white matter bundles: the uncinate fasciculus, connecting to the medial frontal cortex; the cingulum, connecting to the rostral and dorsal ACC; and fronto-striatal fibers, connecting to the NAc, dorsal caudate nucleus, and thalamus. Strong connections of the SCC to the NAc may play a role in the lack of interest and underlying anhedonia characteristic of MDD. The disruption of pathological activity within the SCC by high-frequency stimulation modulates widespread regional brain regions closely connected to the SCC (11).

**Figure 2:**
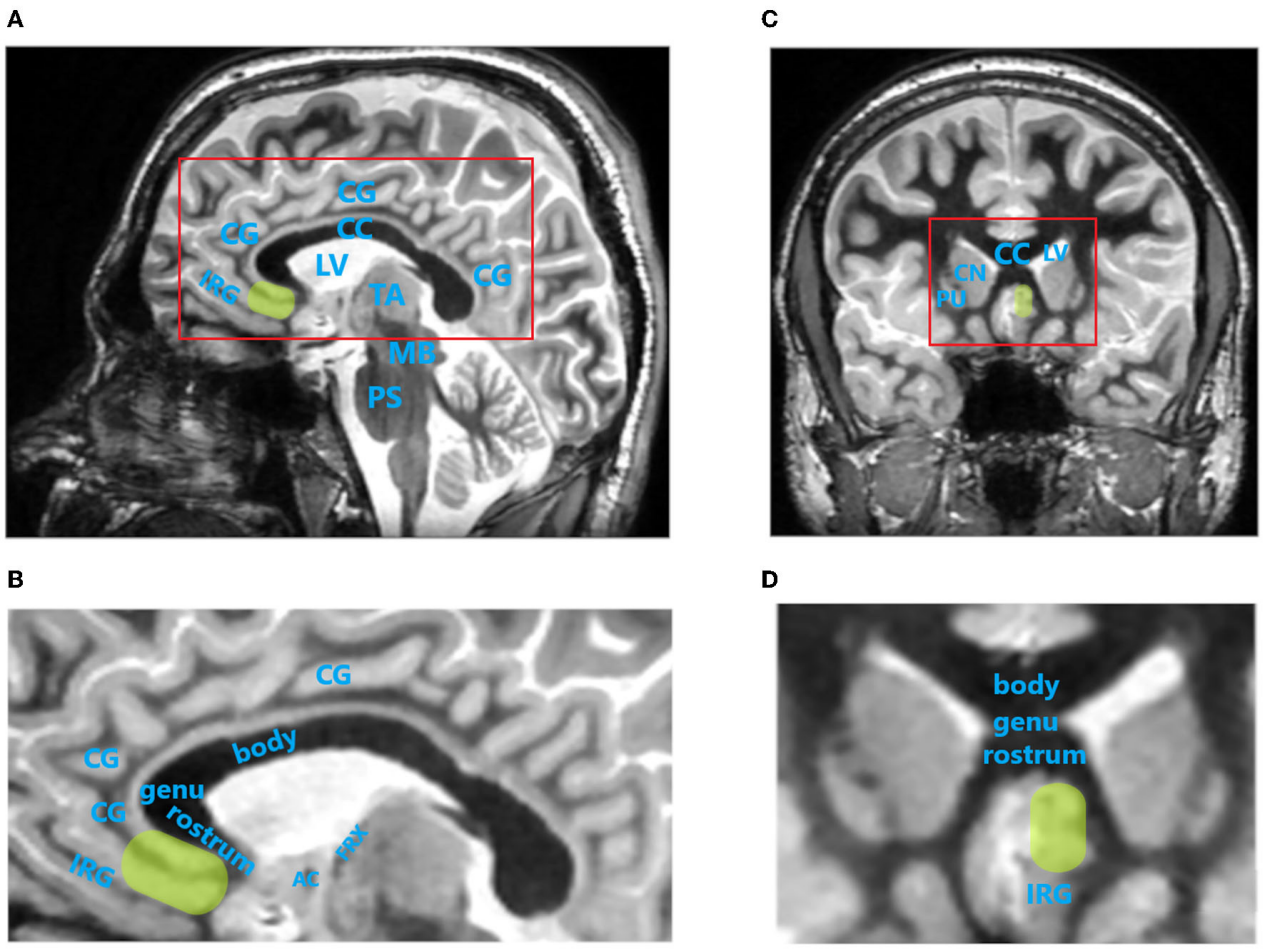
Localization of the subcallosal cingulate cortex (SCC) by magnetic resonance imaging (MRI) using Fast Gray Matter Acquisition T1 Inversion Recovery (FGATIR) sequence. SCC is indicated by green oval region. **(A)**, Paracentral image of sagittal brain MRI scan. Red rectangle indicates brain region magnified in **(B)**. **(B)**, Magnified sagittal MR image of cingulate cortex region. **(C)**, Image of coronal brain MRI scan at the level of SCC. **(D)**, Magnified coronal MR image of cingulate cortex region. AC, anterior commissure; body, body of corpus callosum; CC, corpus callosum; CG, cingulate gyrus; CN, caudate nucleus head; genu, genu of corpus callosum; FRX, ascending column of fornix; IRG, inferior rostral gyrus; LV, lateral ventricle; MB, midbrain; PS, pons; PU, putamen; rostrum, rostrum of corpus callosum; TA, thalamus. MRI images were acquired on 3T SIGNA Architect scanner (GE Healthcare).

The primary aim of SCC DBS for TRD is to reduce activity within this brain region while increasing the activity of dorsal limbic and neocortical regions, especially the dlPFC, dorsal anterior cingulate, posterior cingulate, and premotor and parietal regions, which correspond with clinical improvement in patients with MDD (11, 16, 17). Localization of the SCC by MRI using a Fast Gray Matter Acquisition T1 Inversion Recovery (FGATIR) sequence is shown in Figure 2. The SCC has been marked in yellow color to surrounding brain structures.

### Clinical Trials of SCC DBS for TRD

The first clinical trial of SCC DBS for TRD involved six patients, all of whom met the Diagnostic and Statistical Manual of Mental Disorders (DSM)-IV-TR criteria for MDD (11). Depressive symptoms were evaluated using the Hamilton Depression Rating Scale (HDRS). Clinical response was defined as a >50% decrease in HDRS scores from baseline; clinical remission was defined based on a decrease in HDRS score of eight or less (11). At 6 months of follow-up, four of the six patients were classified as responders, and three patients reached or approximated the criteria for clinical remission (11). All patients experienced dose-dependent, stimulation-related adverse events, such as lightheadedness and psychomotor retardation at high settings (>7.00 V), most often evident at the superior electrode contact. To control a possible placebo effect, patients were blinded to which contact was being stimulated and to the parameter settings. Sham stimulation using zero Volt (sub-threshold stimulation) without was failed to elicit any changes in behavior. This pivotal study presented by Mayberg at al. (11) initiated further studies of SCC DBS for TRD (18–30). The detailed clinical outcomes of open-label studies and RCTs of SCC DBS for TRD are presented in Table 1.

**Table 1:** Open-label clinical trials and randomized clinical trials (RCTs) reporting subcallosal cingulate cortex (SCC) deep brain stimulation (DBS) outcomes for treatment-resistant depression (TRD).

**Authors and year of publication**	***N***	**Study design**	**Follow-up in months**	**Response rate at the last follow-up**	**Remission rate at the last follow-up**	**Adverse events**	**Comments surgical targeting/stimulation settings/stimulation mode/manufacturer**
Mayberg et al. (11)	6	OLS	6	66	50	Skin erosion (1), two skin infection with DBS hardware removal (2)	Local anesthesia, 130 Hz, 60 μs, 3.0–4.5 V, monopolar stimulation, Medtronic
Lozano et al. (18)	20	OLS	12	60	35	Wound infection and DBS hardware removal (3), reinsertion of DBS hardware (1), wound infection managed with antibiotics alone (1), perioperative seizure (1), worsening mood/irritability (2), perioperative headache (4), pain at pulse generator site (1)	This study includes 6 patients from report of Mayberg et al., with 14 new individuals. Local anesthesia 130 Hz, 90 μs, 3.5–5 V, monopolar stimulation, Medtronioc
Lozano et al. (11)	21	OLS	12	62	NA	Suicide (8 weeks after DBS) (1) Suicide attempt (1) Nausea/vomiting (9) Extension malfunction (2) Skin erosion (1) Postoperative headache (6), Persistent pain (4)	Local anesthesia, 128.1 Hz, 93.9 μs, 5.2 mA, St Jude
Puigdemont et al. (20)	8	CRT	24	62.5	50	Cephalalgia (2), neck pain (3), suicide attempt (4 months after DBS) (1)	Local anesthesia, 135 Hz, 90 μs, 3.5–5 V (mean voltage 4.2), bipolar stimulation, Medtronic
Holtzheimer et al. (21)	17	OLS	24	92	58	Device or surgery related Adverse events (8) Suicide attempts (2)	10 patients with MDD, and 7 patients with BP. Local or general anesthesia, 130 Hz, 90 μs, 4–8 mA. St Jude
Merkl et al. (22)	6	OLS	12	NA	30	Postoperative headache, pain, scalp tingling (6)	General anesthesia, 130 Hz, 90 μs, 5 up to 10 V, monopolar stimulation, Medtronic
Choi et al. (23)	9	OLS	NA	NA	NA	NA	Local anesthesia, 130 Hz, 90 μsus, up to 6 V, monopolar stimulation, Medtronic
Holtzheimer et al. (24)	90	CRT	24	48	25	Increase in depressive symptoms (8), infection (6), anxiety (3), suicidal ideation (1), suicide or suicide attempt (1), seizure or convulsion (1), postoperative discomfort (1), hearing and visual disturbance (1),skin erosion (1)	Local or general anesthesia, 130 Hz, 91 μs, 4 mA, monopolar stimulation, St Jude
Riva-Posse et al. (25)	11	OLS	12	81.8	54.5	NA	Local anesthesia, 130 Hz, 91 μs, up to 6 mA. St Jude
Smart et al. (26)	14	OLS	12	78.5	NA	NA	This study includes 11 patients from report of Riva-Posse et al., with 3 new individuals Local anesthesia, 130 Hz, 91 μs, up to 6–8 mA. St Jude (12 patients) 130 Hz, 90 μs, up to 3.5–5 V. Medtronic (2 patients)
Howell et al. (27)	6	OLS	12	33.3	66.7	NA	Local anesthesia, 130 Hz, 90 μs, 4 V, monopolar stimulation, Medtronic
Merkl et al. (28)	8	RCT	24	33	NA	Headache, Pain, Scalp tingling (8), Dizziness; Light postoperative transient (2–4 days) hypomania (8); Inconvenient movement with subsequent hardware explantation (2)	Local anesthesia (3 patients), general anesthesia (5 patients), 130 Hz, 90 μs, 5 up to 7.5 V, monopolar stimulation, Medtronic
Eitan et al. (29)	9	RCT	13	44.4	NA	One serious event, mostly pain and itching at surgical wounds (9)	Local anesthesia,130 Hz, 91 μs, up to 4 mA, monopolar stimulation, St Jude
Ramasubbu et al. (30)	22	RCT	12	23	27	Suicide (1), anxiety and depression, infection with reimplantation (1) Seizures (1)	Local anesthesia,130 Hz, pulse width randomization 90 μs or 210–450 μs, up to 8 mA, monopolar stimulation, St Jude

*OLS, open-label study; RCT, randomized clinical trial; MDD, major depressive disorder; BP, bipolar disorder; NA, not reported; Hz, frequency; us, pulse width; V, voltage; mA, milliampere*.

**Figure 3:**
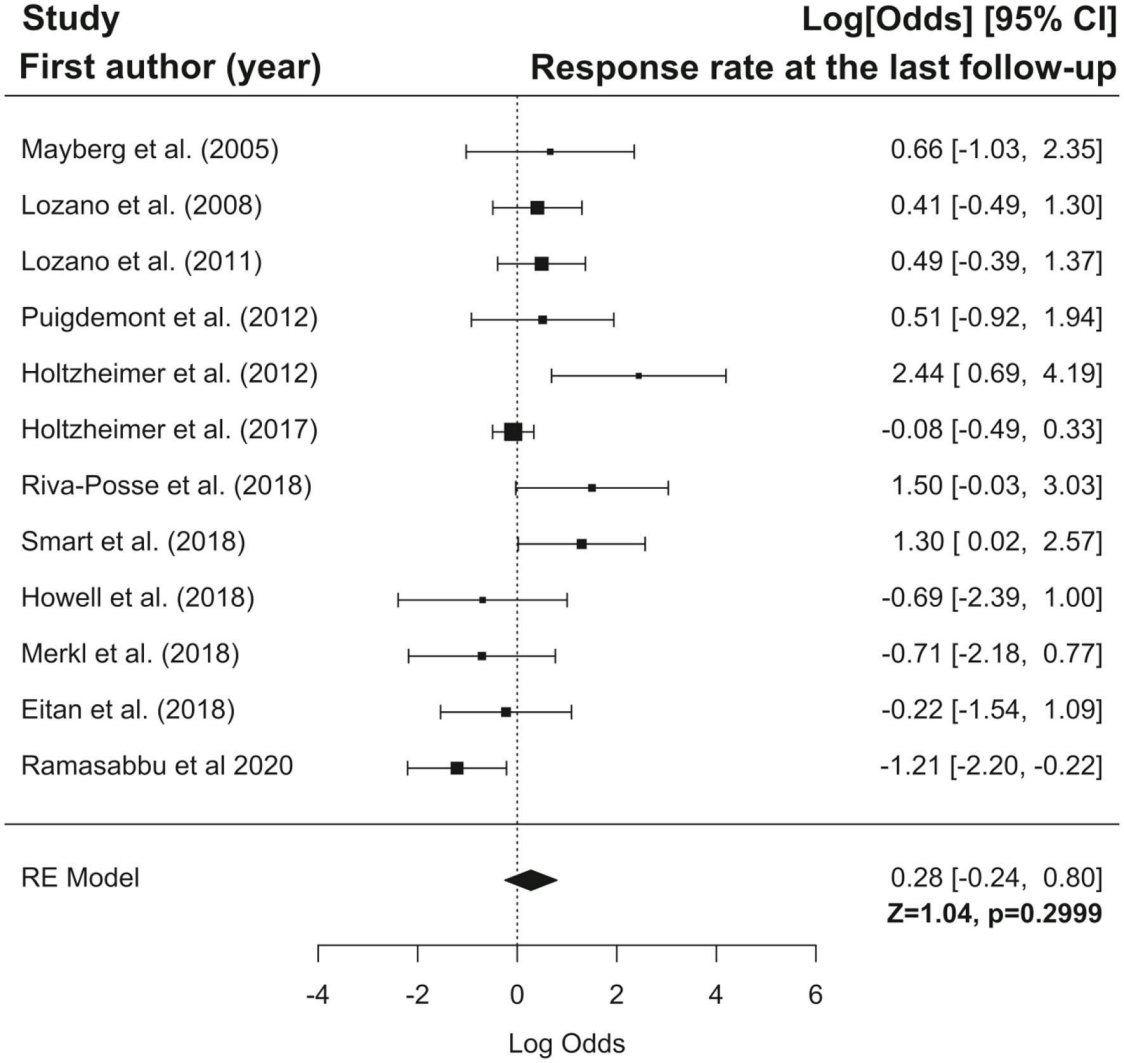
Forest plot showing logit-transformed proportions for response rate at the last follow-up.

**Figure 4:**
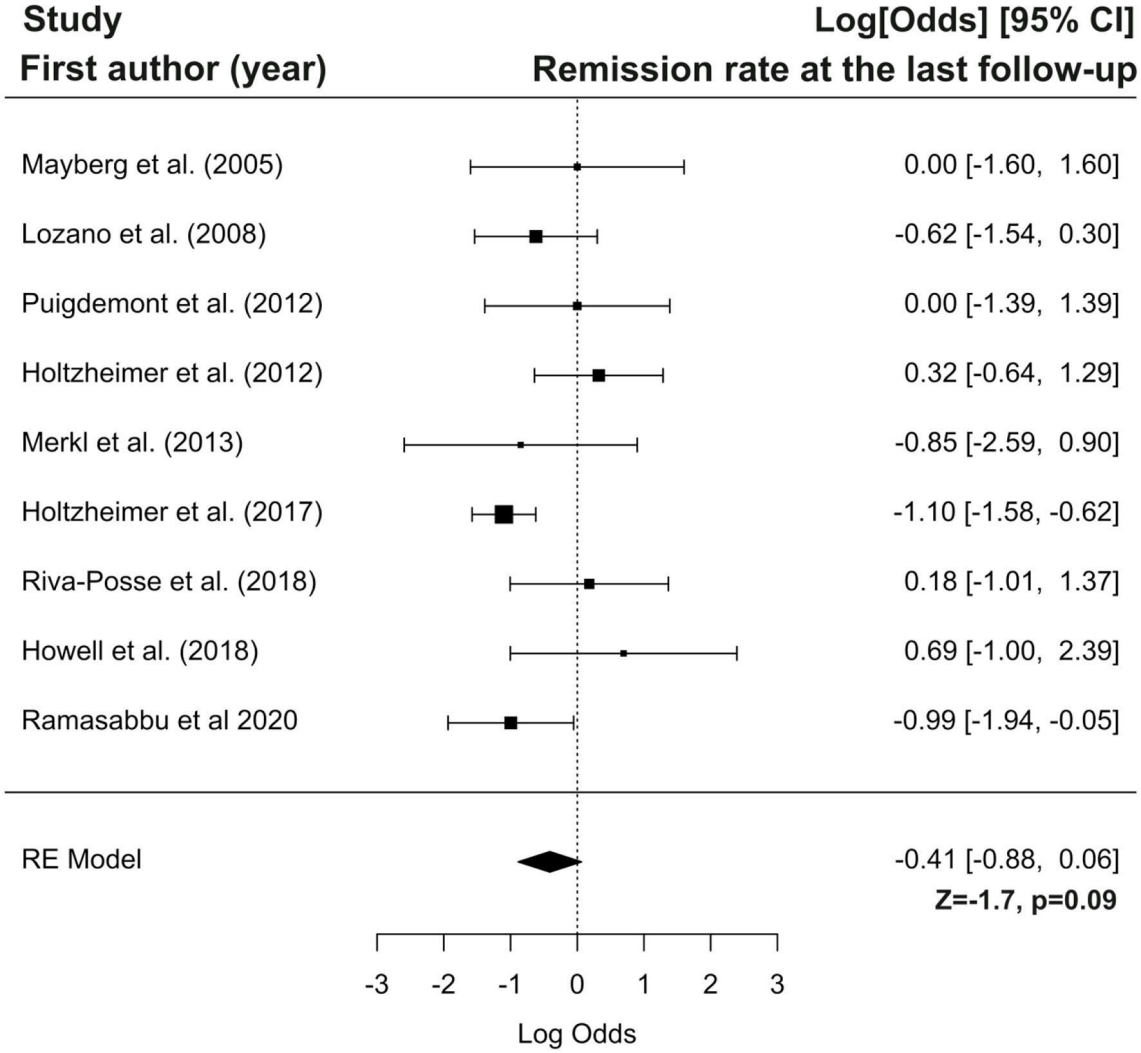
Forest plot showing logit-transformed proportions for remission rate at the last follow-up.

Furthermore we have performed the meta-analysis of selected studies using forest plots. Forest plots present logit transformed proportions of number of patients experiencing the event in respect to the total number of patients from the particular study and 95% confidence interval for response and remission rates at the last follow-up. Black rectangles size are related to precision estimate from a study. Meta-analysis of the logit transformed proportions was conducted using metafor package. Meta-analysis on studies reporting response rate was conducted on data from twelve studies, were this outcome of interest was reported. Based on the results of the test for heterogeneity it was assumed that heterogeneity is significant (I^2^ = 60.76%, Q(df = 11) = 25.03, *p* = 0.009). The estimated logit transformed proportions for response are shown on Figure 3. After transforming to raw scores, proportion for response was equal to 0.57 (with 95% CI: 0.44 to 0.69), Z = 1.04, *p* = 0.2999. Meta-analysis on studies reporting remission rate was conducted on data from nine studies, were this outcome of interest was reported. Based on the results of the test for heterogeneity it was assumed that there is no significant heterogeneity (I^2^ = 42.80%, Q(df = 8) = 13.62, *p* = 0.09). The estimated logit transformed proportions for remission are shown on Figure 4. After transforming to raw scores, proportion for remissions was equal to 0.399 (with 95% CI: 0.2923 to 0.5158), Z = −1.7, *p* = 0.09.

In conclusion, the results of our meta-analysis showed that DBS response and remission rates are not significantly higher than the rate of non-responders and patients without remission, respectively. Presumably, these results might be attributed to the low number of studies, which were characterized by a low sample size and rather high heterogeneity. Therefore, the above conclusions should be treated with caution, and further studies conducted in a larger sample size with a control group are needed.

In the reviewed SCC DBS clinical trials, the response rate was 23–92%, and the remission rate was 27–66.7%, across different time points (11, 18–30). Most studies reporting outcomes of SCC DBS for TRD found that the antidepressant effect was often evident within the first 6 months of DBS (11, 18–30).

Interestingly, a randomized clinical trial (RCT) of SCC DBS for TRD (24) did not demonstrate the positive antidepressant effect evident from other open-label clinical trials (18–30). A randomized clinical study consisting of a double-blind 6-month active vs. sham stimulation phase was stopped after the recruitment of 90 patients due to futility analysis (24). At 6-month follow-up, the response rate in the stimulation arm was 20%, compared to 17% in the non-stimulation arm, leading to study discontinuation (24). Assessment of the open-label active period results revealed that 38 of the 77 patients were classified as responders, and 20 were classified as remitters. The authors highlighted that a poor initial response to DBS might be attributable to multiple factors, such as an extraordinarily long duration of TRD episodes, or a suboptimal contact position for DBS having been chosen (24).

Several studies have tried to improve the accuracy of SCC targeting for DBS procedures (25, 31, 32). The pivotal study by Mayberg et al. (11) utilized PET to guide electrode implantation. In a recent study by Riva-Posse et al. (25), a probabilistic tractography map was used to plan surgical targeting of the SCC in 11 patients with TRD (25). At 12-month follow-up, 82% of the patients with TRD were classified as responders, and 55% were classified as remitters (25). Individualized targeting based on tractography mapping may increase the efficacy of SCC DBS for TRD in future studies (25, 32). Other studies have also implemented probabilistic tractography in SCC DBS targeting (23, 33).

The next step for improving clinical outcomes may be the initiation of closed-loop DBS for psychiatric conditions (34). Experimental and clinical closed-loop DBS systems for Parkinson's disease (PD) are emerging (35). These systems can sense the electrophysiological blueprint of increased beta activity pathognomonic of akinesia and increased muscle rigidity and respond with the delivery of an automatically adapted stimulation (34, 35). Such biomarker-based neurally active implantable pulse generators are commercially available and are utilized mainly for movement disorders, such as PD (36). This approach constitutes a major advance toward improving the outcomes of patients with PD treated with DBS, while simultaneously enhancing our understanding of the pathophysiological mechanisms underlying movement disorders (34–36).

Closing the loop of DBS technology for neuropsychiatric conditions may be more demanding than for movement disorders in clinical practice (34). First, in contrast to movement disorders where stereotactic targets are the subthalamic nucleus (STN) or globus pallidus pars interna (GPi), the most promising targets used for neuropsychiatric indications are fiber tracts connecting widespread disturbed brain areas implicated in conditions, such as TRD and obsessive-compulsive disorder (OCD) (37–40). Second, the prerequisite for closed-loop stimulation may be a well-defined physiologic target with well-recognized neuronal disease-specific brain activity (34). Third, MDD and TRD in particular are heterogeneous disorders, which can present with diverse diagnostic symptoms (2, 34). Importantly, TRD may include undiagnosed comorbid medical conditions, personality disorders, or psychiatric illness with frequently overlapping OCD symptoms (5–8). Fourth, DBS in TRD requires an extended treatment period in order to demonstrate clear clinical outcomes. In contrast, in patients with PD, the clinical effects are visible within minutes. This may impede the identification of biomarkers for specific TRD phenotypes. Even if TRD patients respond to DBS treatment at different time points, the time of recording critical changes needed to define clinical improvement may be missed (34). Repeated recording with a high temporal density over a year or more may be needed for continued DBS treatment. Fifth, there are no programming guidelines for DBS treatment of TRD, and programming is often based on subjective improvements, which are prone to bias (41).

Despite the abovementioned difficulties of closed-loop DBS for TRD, commercially available platforms for longitudinal electrophysiological recording and monitoring are used for neuropsychiatric conditions (34). The time points of clinical improvement suggest that neuroplasticity plays a major role in the efficacy of DBS for TRD. The success of closed-loop DBS in TRD will depend on the identification of symptom-specific biomarkers, which may shed light on causal mechanisms of TRD within the limbic CSTC circuit (34, 41, 42).

### Complications Related to SCC DBS for TRD

Deep brain stimulation-related complications can be divided into three categories, i.e., primarily surgery-related, hardware-related, and stimulation-induced. Surgery-related complications due to SCC DBS are minor, usually transient, and without a profound impact on patient health (11, 18, 22, 24). Erosions with subsequent infections were more common than strictly surgery-related complications (11, 18, 30). The most common complications in TRD patients were stimulation related (18, 19, 24, 28). In cases where the SCC was targeted, patients with TRD experienced suicidal ideation and attempts during follow-up. However, these suicidal events were not considered as being the result of DBS treatment (18, 19, 29, 30).

Patients with TRD referred for SCC DBS constitute a vulnerable group, and close follow-up is indicated in order to reduce fatalities due to attempted suicide. Those who fail to respond to DBS may have an increased risk for suicidal ideation and attempts during follow-up. The inclusion criteria for TRD trials using DBS should be redefined in order to exclude patients with pre-existing suicidal ideation or a history of prior suicidal attempts. It is also important to realize that a favorable response to DBS in patients with TRD does not preclude suicidal attempts during follow-up.

### Alternative Stereotaxic Targets in DBS for TRD

The SCC is the most frequently targeted structure for TRD. The results of SCC DBS are encouraging (18–28). As mentioned above, TRD is a heterogeneous disorder, and the current understanding of its pathophysiology is grounded in a disturbed limbic CSTC circuit (2, 34).

Considering the most common symptoms of TRD, it may be concluded that a target-specific approach for different symptoms profiles is optimal (34). There are three DBS targets within this limbic CTSC loop, which may modulate it in very different ways (18–28, 38, 39). Besides the SCC (Brodmann area 25), the ventral capsule (VC/VS) and medial forebrain bundle (MFB) constitute main stereotactic targets for DBS in TRD (11, 38, 39). Clinical data suggest that anhedonia responds more favorably to MFB DBS and anxiety to VC/VS DBS (38–40). Other structures less commonly used for DBS in TRD are the bed nucleus of the stria terminalis (BNST), lateral habenula (LHb), and the inferior thalamic peduncle (ITP) (37, 42–45).

The selection of these structures is supported mainly by lesional and neuroimaging studies (46). DBS studies themselves have also inspired the use of some targets through tests on animal models or use in humans for other neuropsychiatric illnesses, in which improvement in mood was observed as a positive side-effect (37–39, 43). Some targets have been chosen based on the knowledge of their anatomical and functional significance within neural circuits and neurotransmitter systems implicated in mood disorders (47). These goals were also selected in accordance with the hypothesis of limbic CSTC dysfunction in MDD (12, 13). Since the purpose of this study was to focus on one specific stereotaxic target (SCC), we do not include a comprehensive discussion of the various stereotaxic targets for DBS in TRD, but merely highlight the existence of alternative options. However, we emphasize that the SCC is the most common targeted structure for TRD within the limbic CSTS loop (11).

### Limitations of Existing DBS Studies for TRD

Clinical trials support the efficacy and safety of SCC DBS for severe TRD (11, 18–30). Multiple factors however complicate a comparison across different trials examining SCC DBS in TRD. These include the use of incompatible inclusion and exclusion criteria, the selection of different techniques for targeting the SCC, and different outcomes measures across non-RCTs and RCTs. Earlier SCC DBS studies in TRD have also used different clinical scales with variable follow-up periods. Most prior studies have been open-label, with small sample sizes and failure to control for possible placebo effects. There is also a lack of control sham stimulation period or comparison with controls with the best available medical therapy for TRD (18, 19, 24, 28–30).

Interestingly, an RCT of SCC DBS for TRD failed to support its efficacy, which was ascribed to many factors, which were presumed to have a profound effect on the final clinical outcome (24). The drawbacks of that trial may help to inform the design of future randomized double-blinded trials with a cross-over sham component. First, the optimization period after DBS should be long enough (6 months or more) in order to assess treatment efficacy for TRD. A longer optimization phase may also reduce the strong placebo effect evident in most clinical trials of DBS for psychiatric conditions. The clinical nature of TRD should also be considered that includes its with its waxing and waning clinical symptoms (2, 3). Longer follow-up periods could also enable the determination of more convenient stimulation settings, which may be more specific to the SCC (20, 21). Considering the abovementioned factors, the duration of the optimization phase could even be prolonged beyond 6 months in order to reduce many of the abovementioned confounders.

A limiting factor, which is often forgotten is the implementation of different neurosurgical techniques during DBS lead placement by different surgical teams (18, 20, 25–30). This factor is related to the use of intra-operative micro-recording, macro-stimulation, and awake or asleep surgery during DBS lead placement. TRD is now regarded as a neuronal connectivity disorder. It has been shown that resting-state functional connectivity predicts the success of DBS of distinct anatomical targets (32). The success of DBS may be more related to the engagement of specific neuronal fibers and neuronal circuits compared to specific anatomical coordinates (33). Clinical pre-operative probabilistic tractography has been utilized in SCC targeting (25, 31). This approach has significantly contributed to improved outcomes after SCC DBS for TRD (25). The advantage of using pre-operative tractography by planning surgical targeting is related to selecting responders from non-responders (25, 31). Responders show undisturbed connectivity of a brain structure that is targeted by the DBS lead. This confirms the belief that MDD is a disorder of neuronal brain circuit dysfunction and that effective stimulation depends primarily on the modulation of SCC fibers connecting areas of the brain that are implicated in the pathophysiology of MDD (25, 31, 33).

## Conclusion

SCC DBS for TRD should be regarded as an experimental therapy. The SCC is the most common targeted neuronal structure for TRD. SCC DBS for TRD is a promising new treatment, and up to a third of patients resistant to all other available therapeutic treatment modalities can be substantially helped. Emerging technologies, such as probabilistic tractography, used for SCC targeting may enhance clinical outcomes. In considering the risk for suicidal ideation and attempt, close postoperative monitoring and follow-up are mandatory in this very ill and vulnerable patient population. SCC DBS for TRD should only be administered in the clinical studies driven by experienced multidisciplinary teams.

## Author Contributions

All authors listed have made a substantial, direct, and intellectual contribution to the work and approved it for publication.

## Conflict of Interest

The authors declare that the research was conducted in the absence of any commercial or financial relationships that could be construed as a potential conflict of interest.

## Publisher's Note

All claims expressed in this article are solely those of the authors and do not necessarily represent those of their affiliated organizations, or those of the publisher, the editors and the reviewers. Any product that may be evaluated in this article, or claim that may be made by its manufacturer, is not guaranteed or endorsed by the publisher.
